# Retraction Notice to “Genes Involved in Apoptosis Regulation: Implications for Cancer Therapy”

**DOI:** 10.2174/1389202926999250910152652

**Published:** 2025-09-10

**Authors:** Simone Fulda, Klaus-Michael Debatin

**Affiliations:** 1 University Children’s Hospital, Eythstr. 24, D-89075 Ulm, Germany

The article titled “***Genes Involved in Apoptosis Regulation: Implications for Cancer Therapy***,” published in ***Current Genomics*** (***CG***), has been retracted.

Following a formal complaint and subsequent investigation, it was determined that a significant portion of the content in this review closely resembles an earlier article published online in 2003 (DOI: 10.2174/1570160033378358) authored by the same individuals. This constitutes substantial text recycling or self-plagiarism and violates the journal’s publication ethics.

Such duplication constitutes a serious breach of publishing ethics and is considered plagiarism. In accordance with our policies, this article is hereby retracted from the journal record.

We apologize to our readers and to the original author for any inconvenience caused.

**BENTHAM SCIENCE DISCLAIMER:**

It is a condition of publication that manuscripts submitted to this journal have not been published and will not be simultaneously submitted or published elsewhere. Furthermore, any data, illustration, structure or table that has been published elsewhere must be reported, and copyright permission for reproduction must be obtained. Plagiarism is strictly forbidden, and by submitting the article for publication the authors agree that the publishers have the legal right to take appropriate action against the authors, if plagiarism or fabricated information is discovered. By submitting a manuscript the authors agree that the copyright of their article is transferred to the publishers if and when the article is accepted for publication.

